# Guidelines on pediatric day surgery of the Italian Societies of Pediatric Surgery (SICP) and Pediatric Anesthesiology (SARNePI)

**DOI:** 10.1186/s13052-018-0473-1

**Published:** 2018-03-12

**Authors:** Ugo de Luca, Giovanni Mangia, Simonetta Tesoro, Ascanio Martino, Maria Sammartino, Alessandro Calisti

**Affiliations:** 1 Day Surgery Unit, Santobono-Pausilipon Pediatric Hospital, Napoli, Italy; 2 Department of Anesthesiology, San Camillo Forlanini Hospital, Roma, Italy; 3 Department of Anesthesiology, Perugia University, Perugia, Italy; 4 Pediatric Surgery Ospedale Salesi, Ancona, Italy; 5 Department of Anesthesiology, Policlinico A. Gemelli, Roma, Italy; 6 Pediatric Surgery, San Camillo Forlanini Hospital, Roma, Italy

**Keywords:** Day surgery, Outpatient, Day case surgery, Ambulatory surgery, Guidelines

## Abstract

The Italian Society of Pediatric Surgery (SICP) together with The Italian Society of Pediatric Anesthesia (SARNePI) through a systematic analysis of the scientific literature, followed by a consensus conference held in Perugia on 2015, have produced some evidence based guidelines on the feasibility of day surgery in relation to different pediatric surgical procedures. The main aspects of the pre-operative assessment, appropriacy of operations and discharge are reported.

## Background

Pediatric Day Surgery has become increasingly prevalent in western countries during recent years. Pediatric procedures elegible for day surgery have also been more frequently produced mainly because of the improvement in minimally-invasive surgical techniques, the development of new general anesthetic drugs and the wider use of regional anesthesia. Nowdays, 60% to 80% of operations in a modern pediatric hospital are performed on a day surgery basis. The major advantages of this trend consist in the lessening of psychological stress for children and parents and the reduction in hospital costs, frequency of nosocomial infections and length of surgical waiting lists. In order to evaluate the surgical procedures suitable for day surgery with the same level of reliability as applies in the case of in-patient operations, the Italian Society of Pediatric Surgery (SICP) together with the Italian Society of Pediatric Anesthesia and Resuscitation (SARNePI) have produced EBM-guidelines concerning pediatric day surgery.

## Methods

The literature was examined by means of specific “queries” in the database of NCBI, the National Guidelines Clearinghouse, the Cochrane Library, Medline Complete and Dynamed-Ebsco. The query “ambulatory surgical procedures”[Mesh] and ((“2010/05/01”[PDAT]: “2015/05/31”[PDAT]) and (“infant”[MeSH Terms] or “child”[MeSH Terms] or “adolescent”[MeSH Terms])) provided 391 items, which included 86 clinical trials and 31 reviews.

The Evidence Levels of the proofs and Grade of Recommendation were expressed according to the criteria defined in the Methodological Handbook provided by the National Programme for Guidelines promoted by the Istituto Superiore Sanità (ISS) with the cooperation of the CEVeAS of Modena [1] as reported in Table 1.

**Table 1:** The CEVeAS Scale of the level of evidence of the proofs and the grades of recommendation

LEVEL OF EVIDENCE (LE)
I Evidence obtained from several RCTs and/or reviews of RCT
II Evidence obtained from one RT adequately designed
III Evidence obtained from non randomized cohort studies with case/control or their methanalysis
IV Evidence obtained from case/control retrospective studies or their methanalysis
V Evidence obtained from series of cases without control group
VI Evidences obtained from experts advice, from consensus conferences, etc.
GRADE OF RECOMMENDATION (GR)
A Surgical or diagnostic procedures are strongly recommended because they are sustained by high level scientific evidence, even if not necessarily of type I or II
B It is doubtful that the procedure must be always recommended but it must be carefully considered
C There exists an element of uncertainty both in favor and against the recommendation
D The procedure is not recommended
E The procedure is strongly ill-advised

There are three main stages in day surgery: the pre-operative assessment, the surgical procedure and the discharge.

### Pre-operative assessment

Children have been considered as ideal for day-case management because they usually have little co-morbidity and on account of the fact that many common pediatric operations are well suited for day surgery.

The pre-operative assessment must concern principally:

### Clinical factors

Structured questionnaires completed and signed by the parents and by the pediatrician concerned, covering both social and medical history, are obtained during the pre-operative assessment.

ASA I-II children are suitable for outpatient treatments. ASA III patients are generally excluded, but may possibly be included for low grade surgical procedures.

Premature infants must be excluded if they are of less than 60 weeks post-conception age even if the risk of any post-anesthetic apnoea is related to the grade of prematurity.

Pre-operative investigations (lab tests, x-rays and ECG) are rarely performed according to recent guidelines but may be possibly be requested by the anesthesiologist or by the surgeon during their clinical and anamnestic evaluation. (Table 2).

**Table 2:** Pre-Operative phase

Question	Advise	Evidence	Grading Recomandation	Literature
Family or Social Status excluding Day Surgery	Parents reluctant or unable to take care of the child in the post-operative period at home. Poor domestic hygienic conditions. Lack of a telephone. House more than 1 h travelling distance from an hospital provided with a 24 h emergency facility. Absence of public transport	V	A	[122–127]
Newborns	Full term newborns (Gestional Age Weeks > 38) of less than 1 month are excluded from Day Surgery. Exclusion should be preferably extended to at least 6 months of age. Infants from 2 to 6 months age could be included according to Structure Policy and Surgical Grading.	V	A	[18, 128–131]
ASA III Patients	Normally excluded from Day Surgery. May possibly be eventually included in relation to low surgical grading procedures. There needs to be, at any rate, a prolonged observation post-operatively before discharge.	III	C	[132, 133]
Patient with current Upper Respiratory Infection (URI)	Procedure must be postponed in relation to patients with major respiratory symptoms. If there are mild or moderate symptoms the procedure should be postponed if the child is of less than 1 year of age. In the case of older patients the risk factors should be considered and the appropriacy of the operation assessed in each case.	II	A	[134–136]
Pre-Term	Infants PCA > 60 weeks. Clinically Stable. Anemia corrected.	II	A	[2, 137–140]
Evaluation of Timing	No pre-anesthesia assessment much in advance. An assessment is advisable shortly before the procedure.	V	B	[141–145]
Lab Tests	Routine Lab Tests in healthy patients older than > 1 yr. have a low predictive value	I	A	[146–151]
Medical Records	A parental anamnestic questionnaire is a good tool before any surgical procedure.	IV	C	[152]
Pre-operative Fasting	The administration of clear fluids up to two hours before induction is advised. This lower the residual gastric volume and raise pH.	I	A	[153–156]
Prevention of Nausea and Post-Operatory Vomiting (PONV)	PONV prevention requests a multifactorial approach that includes pre-operative identification of risk factors (family history, age > 3 yrs., Strabismus Repair and ORL surgery). In patients at risk prophylaxis is recommended (i.e.ondansetron 0.05 mg/kg + dexametason 0.015 mg/kg).	I	A	[157, 158]

### Socio-familial factors

A responsible adult, preferably both parents, must be available to transport the child and to provide home assistance in the post-operative period. A telephone must also be available and the home hygiene condition must be satisfactory. Finally a primary care hospital must be accessible within 1 h travelling distance by car from the patient home. All these requirements can be summarized as parental/environmental adequacy.

### Surgical factors

Over recent years the complexity of surgical procedures has increased, with a wider range of children being suitable for day surgery. This is mainly due to the development of minimal invasive surgery and new anesthetic drugs and techniques, the wider use of regional anesthesia and the improved postoperative pain management. Generally speaking, a surgical day-case procedure should last not more than 120 min, without a high risk of post-operative bleeding or uncontrollable post-operative pain.

### Suitable surgical procedures

#### Inguinal hernia and hydrocele

Inguinal hernia and communicating hydrocele are both caused by a failure of obliteration of the processus vaginalis of the peritoneum. The majority of inguinal hernias in infants and children are indirect hernias with direct and femoral hernias only occasionally observed. All these hernias are appropriate for surgical treatment on a day surgery basis either with a traditional open approach **(LE 4; GR A)** or with a laparoscopic approach **(LE 4; GR B)** except for premature infants of less than 60 post- conception weeks of age due to risk of postoperative apnoea **(LE 3; GR E)** [2–22].

#### Undescended testis

A congenital failure of the testis to reach the base of the scrotum after the third month of life requires surgical treatment, preferably if performed between 6 and 18 months of age. In particular, it is advisable to operate within the first year of life in the case of a higher position of the testicle (intrabdominal or intrainguinal) and within 18 months the case of an in extrainguinal lower position (pre-pubic, external inguinal ring or ectopic) (**LE: 3; FR: B)**. A retractile testis does not need medical or surgical treatment, but require regular follow-up until puberty **(LE: 3; GR: A)**. Orchidopexy can be carried out on a day surgery basis [23–29] either by an open technique **(LE: 3; GR: A)** or laparoscopically, in the case of an intra-abdominal testis **(LE: 3; GR: B).**

#### Varicocele

Varicocele is an abnormal dilation of the testicular veins in the pampiniformis plexus, more often (in 90% of cases) of the left side, caused by venous reflux. Indications for surgical treatment consist in testicular hypotrophy (> 20% of the contralateral size) and/or symptomatic varicocele (pain). All operations for the treatment of varicocele are based on the ligation or occlusion of the internal spermatic veins [30–36]. Inguinal or subinguinal microsurgical ligation, anterograd sclero-embolization and suprainguinal ligation, using open or laparoscopic techniques, are all suitable for day surgery **(LE: 5; GR: A/B).**

Indeed, it is safe to say that all inguinal procedures are suitable for day surgery. The low level of evidence in the scientific literature in this field is due to the absence of randomized trials. However this is counterbalanced by the higher grade of recommendation deriving from the widespread and consolidated clinical experience throughout the world in the last 30 years, summarized in certain relevant consensus documents, such as those produced by BAPS and AAPHA) [3–6].

To date, there has been some concern about discharging patients operated laparoscopically on the day of the surgery even in the presence of a good level of evidence, reported in large adult and pediatric series, suggesting the comparative safety of this choice [9, 18, 19, 25, 27, 30, 34]. It would be desirable in the future to plan large multicenter randomized trials to better support the level of evidence of this common practice.

**Table:** 

**SUITABLE FOR DAY SURGERY**	
INGUINAL HERNIAS: **LE: 4**; **FR: A**	
HYDROCELE: **LE: 4**; **FR: A**	
UNDESCENDED TESTIS **LE: 3**; **FR: A**	
VARICOCELE **LE: 3**; **FR: A**	
**TO BE EVALUATED IF SUITABLE FOR DAY SURGERY**	
LAPAROSCOPIC INGUINAL HERNIA **LE: 4**; **FR: B**	
LAPAROSCOPY FOR INTRA-ABDOMINAL TESTIS **LE: 3**; **FR: B**	
LAPAROSCOPIC VARICOCE **LE: 3**; **FR: B**	
**NOT SUITABLE TO DAY SURGERY**	
INGUINAL HERNIA IN PRETERM INFANTS < 60 PCW: **LE: 3**; **FR: E**	

#### Umbilical and alba hernias

Common umbilical hernias after 3 years of life, as well as alba (epigastric) hernias are reported to be well managed as day surgery procedures **(LE: 4; GR: A).**

Rarer huge umbilical hernias (permagna hernias) of the infant must be considered as inpatient procedures because of possible respiratory problems following the reintegration into the abdomen of a large quantity of the bowel.(**LE: 4**; **FR: D**) [37–41].

**Table:** 

**SUITABLE FOR DAY SURGERY**	
UMBILICAL HERNIA (**LE: 4**; **GR: A**)	
ALBA LINE HERNIA (EPIGASTRIC) (**LE: 4**; **GR: A**)	
**NOT SUITABLE FOR DAY SURGERY**	
PERMAGNA UMBILICAL HERNIA IN INFANTS (**LE: 4**; **GR: D**)	

### Phimosis

An absence of the retraction of the foreskin after the first year of life is called phimosis. Only scarring phimosis should be considered as true fhimosis (post-traumatic, due to chronic inflammation or to BXO). Partial or total circumcision is the operation of choice, limiting plastic procedures, like Duhamel’s, only to cases of light preputial stenosis occuring in older children during erection. Both surgical procedures (circumcision and preputial plasty) can be performed as day surgery. (**LE: 5**; **GR: A**) [4–6, 17, 41, 42].

### Buried penis

A buried penis is a normal length shaft enclosed (buried) in prepubic fat. This can be the consequence of obesity, circumcision in an overweight child or the less frequently observed congenital abnormal fixation of the fascia and skin to the balanic sulcus instead of to the base of the penis. Surgical correction is indicated only for the congenital or the post-circumcision forms. **(LE:5;GR:B)** [18, 32, 43–45].

### Webbed penis

An abnormal peno-scrotal junction, resulting in a ventral web, is not only an esthetic problem but it can involve a functional complication during erection. The common V-Y or multiple Z plasty are easily realized as day surgery procedures. (**LE: 5**; **GR: A**) [4, 5].

### Distal Hypospadia

Glandular or distal shaft hypospadias are the most frequent (75%) form of this common uro-genital malformation. Surgical corrections (MAGPI,TIPU,Mathieu) around the 15th month of life are all quite realizable with discharge on the same day either with or without a urethral catheter or stent. Parental adequacy and pain management may suggest the assistance regimen (as an in or outpatient). (**LE: 3**; **GR: B**) [46–52].

**Table:** 

**SUITABLE FOR DAY SURGERY**	
PHIMOSIS AND WEBBED PENIS (**LE: 5; GR: A)**	
**TO BE EVALUATED IF SUITABLE FOR DAY SURGERY**	
BURIED PENIS **(LE: 5; GR: B)**	
DISTAL HYPOSPADIA **(LE: 3; GR: B)**	

### Superficial pathologies

Cysts, nevi and tumors, which reach the fascia or the skull periosteum may be managed on a day surgery basis, as well as embryonal remnants such preauricular sinus or cartilaginous tags. (**LE: 5**; **GR: A**) [6, 15, 17, 18, 53].

### Angiomas and lymphangiomas

Small hemangiomas or lymphangiomas, if susceptible to surgical resection or laser photocoagulation as well as other superficial lumps, may be managed as outpatient procedures. This is also true in the case of larger masses where sclerotherapy is the treatment of choice (**LE: 5**; **GR: A**) [54–59].

### Lymphadenopathy

Superficial lymphadenectomy and sentinel node biopsy can be performed as outpatient procedures (**LE: 5**; **GR: A)** [6, 15, 17, 18, 53].

### Pilonidal disease

The open surgical treatment of pilonidal disease is not recommended for day surgery **(LE: 5; GR: D)** but the primary closure after wide fistulas excision or punch biopsy fistulectomy can be managed as day surgery procedures **(LE: 5; GR: B)** [60–64].

**Table:** 

**SUITABLE FOR DAY SURGERY**	
NEVI, TEGUMENTAL AND EPIFASCIAL LUMPS **(LE: 5; GR: A)** SURGICALLY RESECTABLE OR SCLEROSABLE HEMANGIOMA AND LIMPHANGIOMA **(LE:5:GR:A)** SENTINEL NODE BIOPSY AND SUPERFICIAL LIMPHADENECTOMY **(LE: 5; GR: A)**	
**TO BE EVALUATED IF SUITABLE FOR DAY SURGERY**	
PILONIDAL DISEASE PRIMARY CLOSURE OR PUNCH FISTULECTOMY **(LE: 5; GR: B)** PILONIDAL DISEASE OPEN TREATMENT **(LE: 5; GR: D)**	

### Ankyloglossia (tongue-tie)

Tongue-tie requires frenulotomy if it interferes with suction or causes speech problems. Within the first six-nine months frenulotomy can be carried out as office surgery.In relation to older infants a light and short general anesthesia, feasible as day surgery, is required **(LE: 5; GR: A)** [65–67].

### Diastema

An excessive gap between superior teeth is called a distema and is frequently observed when the superior labial frenum is hypertrophic and inserted on the free edge of the gums. In this case, and mainly for esthetic reasons, a z plasty correction can be realized when the permanent dentition has been completed. **(LE:5;GR:A)** [6, 15, 17, 18, 53].

### Mucocele

Mouth floor, labial and sublingual mucoceles are all resectable as outpatient procedures. Larger ranulas are more frequently treated with marsupialisation to prevent relapse. **(LE:5;GR:A)** [6, 15, 17, 18, 53].

### Cleft lip

Several studies with good scientific evidence report the feasibility of cleft lip and anterior palate surgical correction with a day surgery regimen. **(LE:3;GR:B).**

The decision depends mainly on the experience of the surgeons and their familiarity with these procedures [68–75].

**Table:** 

**SUITABLE FOR DAY SURGERY**	
TONGUE TIE **(LE: 4; GR:A)** DIASTEMA **(LE: 5; GR:A)** MUCOCELE **(LE:5;GR: A)**	
**TO BE EVALUATED IF SUITABLE FOR DAY SURGERY**	
CLEFT LIP AND PALATE **(LE: 3; GR: B)**	

### Branchial anomalies

Sinuses, cysts and fistulas of the second and third branchial arch can be excised as outpatient procedures **(LE: 5; GR:A)** whereas the resection of the first and fourth branchial arch cysts must be evaluated for overnight stay in hospital. **(LE: 5; GR: B)**. Partial thyroidectomy is often necessary to remove a cyst of the 4th branchial arch and therefore an overnight stay in hospital is recommended **(LE: 5; GR: C**) [6, 15, 17, 18, 53, 76–78].

### Thyroglossal cyst

The Sistrunk procedure involves the excision of the cyst or fistula together with the body of the hyoid bone and the suprahyoid duct as far as the foramen caecum. An accurate hemostasis during the procedure is essential and, when this rule is correctly applied the patient can be safely discharged on the day of the operation. This decision should be made taking into account the surgeon’s experience and the duration and difficulty of the operation. **(LE: 4; GR:B**) [79–81].

**Table:** 

**SUITABLE FOR DAY SURGERY**	
2ND AND 3RD BRANCHIAL ARCH SINUSES, CYSTS AND FISTULAS **(LE**: **5; GR: A)**	
**TO BE EVALUATED IF SUITABLE FOR DAY SURGERY**	
1ST AND 4TH BRANCHIAL ARCH CYSTS **(LE**: **5; GR: B)** THYROGLOSSAL DUCT CYSTS **(LE**: **4; GR: B)** PARTIAL THYROIDECTOMY **(LE**: **5; GR: C)**	

### Appendicitis

Despite a good level of evidence in both adult and pediatric scientific literature reporting, the feasibility of non complicated appendectomies or interval appendectomies in day surgery, there is still uncertainty among surgeons about discharge on the day of surgery in such cases **(LE: 3; GR:B**) [82–87].

### Gallbladder diseases

Gallbladder diseases in children are quite rare particularly if compared to adults. Laparoscopic cholecystectomy is the gold standard of treatment. The widespread diffusion of this procedure has produced a great number of high quality scientific studies (RCT) reporting the feasibility and safety of this procedure in day surgery.

In the pediatric field laparoscopic cholecystectomy is less frequently performed with a consequent reduction in the surgeon’s experience and confidence. For this reason the pediatric consensus conference held in Perugia, despite the high quality of evidence **(LE: 1),** preferred to assign a grade of recommendation B to this procedure in day surgery **(LE: 1; GR: B**) [88–98].

### Gastric Funduplication

Currently, gastric fundoplication is mainly performed laparoscopically both in adults and children. The degree of post-operative pain has been reduced together with the duration of hospitalization. A number of good evidence based studies have been reported in adult and pediatric scientific literature suggesting the feasibility of this procedure as day-case surgery with early postoperative feeding and domiciliary pain control. However, as in the case of cholecystectomy, due to the limited use of this practice in pediatric surgery, discharge on the day of fundoplication is not yet recommended except in relation to centers with considerable experience. **(LE: 4; GR: C**) [99–101].

### Gastrostomy

PEG (percutaneous endoscopic gastrostomy) is the gold standard to ensure enteral nutrition in neurologically impaired children. Sometimes, when PEG is inadvisable, a MAG (microlaparoscopic assisted gastrostomy) is preferred. Both procedures have been reported as suitable for day surgery, but the consensus conference proposed a more prudential approach if the surgeon is not well experienced with these procedures **(LE: 3; GR: C)** [102–108].

**Table:** 

**SUITABLE FOR DAY SURGERY**	
APPENDECTOMY **(L: 3; G.R.: B)** CHOLECYSTECTOMY **(L: 1; G.R.: B**)	
**TO BE EVALUATED IF SUITABLE FOR DAY SURGERY**	
GASTRIC FUNDOPLICATIO **(L: 4; G.R.: C)** GASTROSTOMY **(L: 3; G.R.: C)**	

### Pyeloplasty

As for the previously mentioned more complex procedures, pyeloplasty has been reported to be suitable for a day surgery regimen either when performed with an open or laparoscopic approach. Additionally in this case the level of evidence is fairly good (LE: 4). However, the consensus conference suggestion is that careful consideration should be given and the day surgery approach should be adopted only by very experienced team. **(LE: 4; GR: C)** [109–114].

### Vesico-ureteric reflux

The endoscopic subureteric injection of bulking materials is the most popular mini-invasive treatment for vesico-ureteric reflux. Many surgeons perform this procedure on an outpatient basis. **(LE: 4; GR: B**) [114].

### Nephrectomy

For many years nephrectomies of non-functioning kidneys or kidneys containing masses have been reported both in adults and children. The nephrectomy is most often carried out by retroperitoneoscopy or laparoscopy. In this case, day surgery management is limited to very experienced centers.In other cases in-patient admission is recommended. **(LE: 5; GR: C**) [110, 115–117].

**Table:** 

**SUITABLE FOR DAY SURGERY**	
VESICO-URETERIC REFLUX **(L: 4; G.R.: B)**	
**TO BE EVALUATED IF SUITABLE FOR DAY SURGERY**	
PYELOPLASTY **(L: 4; G.R.: C)** NEPHRECTOMY **(L: 5; G.R.: C)**	

#### Anesthesia

The aim of anesthesia is to provide a rapid smooth induction, good operation conditions and prompt recovery. Post-operative pain coverage is also desirable. The laryngeal mask airway is generally used for both spontaneous and controlled ventilation. Tracheal intubation is necessary for laparoscopic procedures and neck procedures. Local anesthesia may be planned with good local anesthetic techniques and pre-operative counseling [118].In the intra-operative phase we have compared two general anaesthesia techniques and examined the role of caudal anaesthesia in post-operative analgesia (Table. 3).

**Table 3:** Intra-operative phase

Question	Advise	Evidence	Grading Recomandation	Literature
*General Anesthesia (Inhalation Anaesthesia* vs. *TIVA)*	No differences between the two techniques have been observed in causing PONV, emergence agitation and respiratory and hemodynamic complications, and in influencing the length of stay in the recovery unit.	***I***	***A***	[159]
*Caudal Anesthesia and post-operative analgesia*	Of all the loco-regional techniques, caudal block has shown the best results in the short and long term, although maintaining a significant risk of motor block and urinary retention	***I***	***A***	[160–165]

#### Discharge

Recovery from an operation depends on several factors: the duration of the operation, site of the surgery, anesthetic technique employed and age of the patient. Ped-PADDS is a score system adapted for pediatric patients (Table. 4) [119–121]. As soon as the patient has met the discharge criteria (a score of 9/10) he/she may be discharged with written directions for home assistance and telephone numbers on-call 24/24 h. A clinical report provided to pediatrician concerned is also recommended.

**Table 4:** Discharge phase

Question	Advise	Evidence	Grading Recomandation	Literature
Discharge (Ward to Home)	The Ped-PADSS score system was evaluated and found to be s simple, practical and suitable. It can also improve the patient flow thus reducing the duration of hospitalization.	***V***	***A***	[119–121]

## Conclusions

These guidelines are based on a review of the literature in relation to different aspects of day surgery including enrollment or exclusion criteria, the surgical feasibility of the most common pediatric operations, customer satisfaction, the safety of day surgery, discharge criteria have all been reported and scored according to evidence based scientific proofs.

For more more than a century pediatric day surgery has been is carried out as the best practice for several common pediatric surgical procedures. However, still today, there are no good proofs supporting the feasibility of the most common pediatric day case surgery procedures on account of the absence of well designed randomized trials. Nevertheless, the widespread worldwide experience in relation to these routine operations suggests that they should be assigned a high score in the grade of recommendations scale.

The goal of these guidelines is to provide pediatric surgeons with a broader range of pediatric operations feasible in a day surgery setting with the same degree of safety as that ensured in relation to in-patient operations. The well known advantages of day surgery consist in the reduction in hospital infections, the lessening of psychological stress, the higher level of customer satisfaction, the shortening of waiting lists and the reduction in hospital costs.
